# Female asylum seekers with musculoskeletal pain: the importance of diagnosis and treatment of hypovitaminosis D

**DOI:** 10.1186/1471-2296-7-4

**Published:** 2006-01-23

**Authors:** G de Torrenté de la Jara, A Pécoud, B Favrat

**Affiliations:** 1 Medical Outpatient Clinic, University of Lausanne, Switzerland

## Abstract

**Background:**

Hypovitaminosis D is well known in different populations, but may be under diagnosed in certain populations. We aim to determine the first diagnosis considered, the duration and resolution of symptoms, and the predictors of response to treatment in female asylum seekers suffering from hypovitaminosis D.

**Methods:**

Design: A pre- and post-intervention observational study.

Setting: A network comprising an academic primary care centre and nurse practitioners.

Participants: Consecutive records of 33 female asylum seekers with complaints compatible with osteomalacia and with hypovitaminosis D (serum 25-(OH) vitamin D <21 nmol/l).

Treatment intervention: The patients received either two doses of 300,000 IU intramuscular cholecalciferol as well as 800 IU of cholecalciferol with 1000 mg of calcium orally, or the oral treatment only.

Main outcome measures: We recorded the first diagnosis made by the physicians before the correct diagnosis of hypovitaminosis D, the duration of symptoms before diagnosis, the responders and non-responders to treatment, the duration of symptoms after treatment, and the number of medical visits and analgesic drugs prescribed 6 months before and 6 months after diagnosis.

Tests: Two-sample *t-*tests, chi-squared tests, and logistic regression analyses were performed. Analyses were performed using SPSS 10.0.

**Results:**

Prior to the discovery of hypovitaminosis D, diagnoses related to somatisation were evoked in 30 patients (90.9%). The mean duration of symptoms before diagnosis was 2.53 years (SD 3.20). Twenty-two patients (66.7%) responded completely to treatment; the remaining patients were considered to be non-responders. After treatment was initiated, the responders' symptoms disappeared completely after 2.84 months. The mean number of emergency medical visits fell from 0.88 (SD 1.08) six months before diagnosis to 0.39 (SD 0.83) after (P = 0.027). The mean number of analgesic drugs that were prescribed also decreased from 1.67 (SD 1.5) to 0.85 (SD 1) (P = 0.001).

**Conclusion:**

Hypovitaminosis D in female asylum seekers may remain undiagnosed, with a prolonged duration of chronic symptoms. The potential pitfall is a diagnosis of somatisation. Treatment leads to a rapid resolution of symptoms, a reduction in the use of medical services, and the prescription of analgesic drugs in this vulnerable population.

## Background

Deficiency of vitamin D, leading to osteoporosis and osteomalacia, is well known in elderly people in Western countries. A European survey in community dwellers over 70 years showed hypovitaminosis D (25- [OH] vitamin D <30 nmol/l) in 36% of men and 47% of women [1].

Hypovitaminosis D and osteomalacia have also been reported in the immigrant Indo-Asian population in the UK since the 1960's [2-5]. Yet this disorder remains widely underconsidered according to more recent studies, with one reporting a mean period of 59.2 months of complaints before the diagnosis was established [6], and another reporting a prevalence of 78% of hypovitaminosis D (versus 58% in controls) in an Indo-Asian population attending a rheumatology clinic in the UK [7].

A recent study in the United States showed that of 150 consecutive patients (immigrant and non-immigrant) of a community clinic with persistent, non-specific musculoskeletal pain, 100% had vitamin D insufficiency (<50 nmol/l) and 28% had severe deficiency (<20 nmol/l) [8]. We have also previously reported eleven cases of symptomatic hypovitaminosis D in female asylum seekers [9].

The condition has also been assessed in consecutively admitted medical inpatients: 57% were considered vitamin D deficient (<37.5 nmol/l), of whom 22% were severely deficient (<20 nmol/l) [10].

Finally, in recent years, several studies have examined the prevalence of hypovitaminosis D in healthy subjects [11-13]. In the late winter months, it appears that 36% of young healthy adults in Boston suffer from the condition (25-(OH) vitamin D <50 nmol/l) [13] as well as 34% of healthy adults in Brussels (25-(OH) vitamin D <40 nmol/l) [12].

Thus, the disease is more prevalent than we generally suspect, not only in at-risk individuals in our multicultural societies, but probably also in a population that appears less vulnerable.

Concerning treatment, the benefit of the prophylactic use of vitamin D and calcium has been established in Asian immigrants in the UK [14], as well as in elderly subjects in regard to its effect on fractures and falls [15,16]. Hardly any studies have measured the impact or clinical response to treatment over time in a younger immigrant population. We are aware of one study, on osteomalacic myopathy in veiled Arabic women in Denmark with a mean age of 32.2 years, that demonstrated a normalisation of muscle strength (except in maximal voluntary contraction) at 6 months after initiation of treatment [17].

The purpose of the study was to assess the impact of diagnosis and treatment in female asylum seekers with hypovitaminosis D. We first noted the diagnosis made by the primary care physicians, as well as the delay in the establishment of the diagnosis of hypovitaminosis D. We then assessed the response to treatment by these parameters: improvement of clinical symptoms (bone pain, muscle weakness, and fatigue), variation in the number of analgesic drugs prescribed, and number of medical visits before and after treatment.

Concerning the response to treatment, no predictors have yet been defined. We therefore tried to extract from our study variables such as age, length of stay in Switzerland, number of chronic illnesses, and existence of psychiatric comorbidity as possible predictors of a positive response to treatment.

## Methods

### Patients

The study investigated female asylum seekers attending an academic primary care centre serving a population of 100,000 between March 2000 and April 2002. All of the patients belong to a health network with a nurse practitioner gate-keeping system and primary care physicians. The patients must always consult within this setting and cannot change their primary care physician without informing the nurse practitioner. After a certain number of cases of hypovitaminosis D were diagnosed in 2000, the centre's primary care physicians were informed by two circular letters, in March and April 2001, of the suspected high prevalence of this disease in female asylum seekers, particularly those with a minimal exposure to sunlight and presenting with a history of bone pain, proximal muscular weakness, a change in gait and/or fatigue. In suspected cases seen within the outpatient department (emergencies and follow-ups), we checked serum levels of 25-(OH) vitamin D, while the physician determined other biochemical parameters if judged necessary: calcium, phosphate, alkaline phosphatase, and parathyroid hormone (PTH). We included women with symptoms of hypovitaminosis D and a confirmed deficiency in the study. Only women were included because of their risk factors and histories. There were no exclusion criteria. In our setting, we did not constitute a control group. The physicians were asked several times to report their cases, but we did not revise all files systematically. The physicians were advised to follow treatment recommendations specified in the circular letters (300,000 IU of intramuscular cholecalciferol with an ongoing course of 800 IU of cholecalciferol associated with 1000 mg of calcium), but were free not to do so. Therefore, the decisions to dose 25-(OH) vitamin D, to report the cases, and to treat, if necessary, the patients, were left to the physicians who were directly in charge of the patients. Nevertheless, we discussed the two circular letters extensively with the primary care physicians and had informal discussions as well.

No approval from a ethics committee was required for this study. Indeed, in Switzerland, a chart review does not need an ethical committee acceptance. All the patients included in the study gave their consent concerning the treatment that is appropriate for hypovitaminosis D. Furthermore, the physicians were free to follow treatment recommendations.

### Study design and treatment

The study was a prospective observational study. Guidelines for treatment were suggested in the circular letters and the majority of patients received two intramuscular injections of 300,000 IU of cholecalciferol at monthly intervals, as well as an ongoing course of oral calcium (1000 mg) and cholecalciferol (800 IU = 20 μg). After 6 months follow-up, we reviewed the medical and nurse practitioner records to determine the main outcomes. There was no standardized questionnaire designed for the follow-up. The information was retrieved from the files with an extraction sheet only.

25-(OH) vitamin D was measured by a radioimmunoassay (RIA) with an ^125^I-labelled tracer (DiaSorin Inc.). Calcium and phosphate levels were measured by spectrophotometry (Roche). The reference ranges are 21–131 nmol/l for 25-(OH) vitamin D, 2.15–2.55 mmol/l for calcium and 0.8 – 1.6 mmol/l for phosphate. (For 25-(OH) vitamin D, 1 μg/l = 2.5 nmol/l). The reference range for 25-(OH) vitamin D is derived from a group of 20 male and 24 female healthy, predominately Caucasian volunteers from the midwestern USA, aged between 23 and 67 years, during the month of October (DiaSorin Inc.). It is well known, however, that this reference range describes a severe hypovitaminosis D, and that levels below 50 nmol/l are considered insufficient (see Discussion section).

### Outcomes

We determined a number of parameters, including region of origin, length of stay in Switzerland, first diagnosis, number of months prior to diagnosis, number of chronic illnesses, psychiatric comorbidity, number of visits and emergency visits six months before and six months after diagnosis, number of analgesic drugs prescribed 6 months before and 6 months after diagnosis, resolution of symptoms after treatment (we defined two categories: complete resolution of symptoms as responders and partial or no resolution as non-responders) and duration of resolution (partial or complete).

### Statistics

Two-sample *t-*tests, chi-squared tests, and logistic regression analyses were performed. Analyses were performed using SPSS 10.0.

## Results

The study population comprised 18 (54.5%) Somali women, 12 (36.4%) Balkan women, and 3 (9.1%) women of other origin. The mean age was 38.8 years (SD 16.9). Twenty-three (69.7%) of the women wore a veil (partial or complete, including gloves), and no corresponding details were available for 2 patients (6.1%). The population had been residing in Switzerland for a mean period of 5.27 years (SD 3). The patients were suffering from a mean of 2.76 chronic illnesses, such as, iron deficiency with or without anaemia, obesity, gastritis, tension headache, and hypertension (SD 1.97), and 10 patients (30.3%) were considered to have one or more psychiatric diagnoses other than somatisation.

The first diagnoses, before hypovitaminosis D was considered, were chronic back pain, generally associated with pelvic or rib pain, in 17 patients (51.5%); somatisation disorder in 7 patients (21.2%); and multiple unexplained somatic symptoms in 6 patients (18.2%). Regrouping these different diagnoses, we obtain a total of 90.1% of patients with an initial diagnosis related to somatisation. In 3 patients (9.1%), the diagnosis of hypovitaminosis D was mentioned initially. The physicians treating these 3 new patients had been formerly informed of the possible high prevalence of the disease prior to the consultation and had suspected it on presentation.

The mean duration of symptoms before diagnosis was 2.53 years (SD 3.20). The duration of symptoms after seeing a physician in our centre was 1.87 years (SD 2.20). Nevertheless, in retrospect, the majority of complaints were quite typical of hypovitaminosis D from the outset.

On diagnosis, the mean serum 25-(OH) vitamin D level was 11.32 nmol/l (SD 4.55). Most of these measurements (87.9%, 29 patients) were made from November to May, at a time when sunshine levels are low (latitude 46.3°).

For 28 patients (84.8%), the mean blood calcium level on diagnosis was 2.17 mmol/l (SD 0.09), and 42.9% of these patients had hypocalcaemia (<2.15), with a minimum of 1.92 mmol/l.

Phosphate concentrations were measured in 25 patients (75.7%), and the mean level was 0.98 mmol/l (STD 0.31). Of these patients, 32% were hypophosphataemic (<0.8) and none were hyperphosphataemic.

Treatment consisted only of calcium and cholecalciferol p.o. in 10 patients (30.3%). The remaining 23 received, in addition, the two injections of cholecalciferol.

With regard to symptom resolution, 22 patients (66.7%) experienced complete resolution and 11 (33.3%) presented either partial (n = 6) or no resolution (n = 5). The beginning of symptom resolution in responders was already noted at 1.47 months (SD 0.59), and resolution was complete at 2.84 months (SD 1.76). One patient required seven months of treatment to be free from symptoms.

Three patients did not consult the primary care physician or the nurse practitioner in relation to the symptoms of hypovitaminosis D after initiation of treatment. They were considered to be responders to treatment, since the medical facilities were totally accessible to them and, on the two occasions on which they did consult the nurse, they did not complain of their initial symptoms.

During the whole period preceding diagnosis, the patients received a mean of 3.27 analgesic drugs (SD 2.28), compared to a mean of 1.67 (SD 1.51) during the 6 months before diagnosis. In the 6 months after diagnosis and initiation of treatment, the number of analgesic drugs fell to 0.85 (SD 1), (P = 0.001). Certain patients suffered from chronic medical conditions (tension headache, osteoarthritis, irritable bowel syndrome, migraine) requiring analgesic medication that did not vary significantly over the 12-month period for which we analysed the data.

We analysed the follow-up visits and the emergency visits. Our centre was open 24 hours a day, 7 days a week. After diagnosis, 27 patients (81.8%) received a medical follow-up in our centre, 3 patients (9.1%) changed physician after at least one medical visit, and the remaining 3 (9.1%) were followed up by the nurse practitioners. There was no difference between the follow-up visits before and after diagnosis. However, the emergency visits fell from 0.88 (SD 1.08) six months before diagnosis to 0.39 (SD 0.83) six months after (P = 0.027).

In a univariate analysis we analysed age, length of stay in Switzerland, number of chronic illnesses, psychiatric comorbidity, presence of a veil, level of 25-(OH) vitamin D, and duration of symptoms before diagnosis, in relation to the resolution of symptoms. None of these variables was significant. According to our logistic regression analysis, neither age, nor the number of chronic illnesses, nor the existence of psychiatric comorbidity were predictors of the response to treatment.

## Discussion

Our results suggest that hypovitaminosis D is still going undiagnosed in immigrant women. Indeed, the first diagnosis evoked, in an often psychologically difficult context, is one suggestive either of somatoform disorder as described in the International Classification of Diseases, 10th revision [18] or "somatisation" as defined in the study of Simon et al [19]. In this study, we refer particularly to the definition concerning patients with psychological disorders who report multiple unexplained somatic symptoms. Nevertheless, pain due to hypovitaminosis D is quite well defined. It is felt in the bones, not in the joints. In general, it is symmetrical, beginning in the lower back and then spreading to the pelvis, upper legs, and ribs. The patients may also present with proximal muscle weakness.

In our study, the complaints lasted for a considerable length of time, with important psychosocial repercussions in an already vulnerable population. This reflects the poor knowledge of hypovitaminosis D prevalent in a group of Swiss primary care physicians, and confirms the study by Nellen [6]. The impact of information is important, leading in our study to the diagnosis of 33 new cases in a year and a half. We wish to emphasize the importance of the diagnosis in a female Balkan population, a group in which osteomalacia has not yet been formally described. Only one-third of these patients wore a veil, so there is no direct correlation between these two parameters. It is nevertheless possible that global sun exposure (due to housebound status) is limited, and that dietary factors have an influence in this population.

After treatment, subjective improvement was already present after a mean of 1.47 months, and resolution was complete after a mean of 2.84 months, with complete resolution at a maximum of 7 months in all patients who responded to treatment. The resolution of symptoms is known to occur approximately between 3 and 6 months: 3 months for symptoms due to the osteopathy [6] and 6 months for the myopathy [17].

Our results also show a reduction in the use of medical services and the prescription of analgesic drugs. These results should be considered preliminary, because no control group was constituted. Nevertheless, these two facts could be of primary importance. The reduction in emergency visits, not necessarily linked to hypovitaminosis D, probably reflects a general improvement in well-being, compared to periods when the patients were suffering from chronic pain. This has not been evaluated, but there may have been an improvement in psychological factors that elevated the threshold for an emergency medical visit. Secondly, the reduction in the prescription of analgesic drugs is probably paralleled by a reduction in use, as all NSAIDs in Switzerland are obtained on prescription and the population studied possesses limited financial resources, which limit its access to over-the-counter drugs. The probable reduction in use also reduces the potential harmful side effects, particularly those associated with NSAIDs. The economic impact of these two results has not been established, but is another interesting consideration.

In the studied population, we found very low levels of 25-(OH) vitamin D, which is regarded as the best laboratory indicator of functional vitamin D status [20]. Over the past few years, this status has been regaining considerable attention. Vitamin D receptors are found not only in bone and muscle but also in the breast, colon, prostate, immune system, brain, and probably other tissues of the body. Vitamin D deficiency affects bone metabolism, and results in osteoporosis and osteomalacia (or rickets, in children), and evidence is now emerging that hypovitaminosis D has other important adverse effects on health, such as increasing the risks of autoimmune diseases, cancers, and other chronic affections [21-23]. A long-term study of 180,000 women showed that women taking 400 IU of vitamin D daily had 40% less risk of developing MS than those who did not [24]. The risks of prostate and colon cancer are lower if 25-(OH) vitamin D levels are above 50 nmol/l [25-27]. In this context, the optimal levels of vitamin D for health benefits have been subject to a number of discussions and studies. It is now widely accepted that 25-(OH) vitamin D levels below 20 nmol/l are indicative of severe deficiency [28]. Levels of at least 50 nmol/l [29], and according to some authors, 78 nmol/l [30], are necessary to prevent secondary hyperparathyroidism. Recent evidence showed that calcium is malabsorbed and fracture risk increases at serum levels below ~80 nmol/l [31]. A 25-(OH) vitamin D level between 80 and 125 nmol/l seems optimal for general health [21,22,31]. Risk factors (reduced exposure to sunlight and strict vegetarian diet) [10,32,33] must be minimized to achieve these concentrations. Nevertheless, these risk factors are difficult to modify; even in Australia, the paradox of hypovitaminosis D in a sunny country is emerging as a public health problem [34]. The most cost-effective way to reach optimal levels of 25-(OH) vitamin D is increasing UVB exposure, by exposing the hands, arms, and face, if culturally acceptable and with caution in low latitudes, without sunscreen for 5–15 min between 1000 and 1500 h in the spring, summer, and fall for individuals with type II and III skin. Diet does not provide high doses of vitamin D, since very few foods contain it (oily fish being one) and fortified food has not met public health expectations. Routine supplementation could be the only effective way of preventing hypovitaminosis D in the population described in our study, since it is not likely that sun exposure habits and diet will change to any meaningful extent. For example, a large educational campaign within the Asian community of Rochdale, UK, between 1970 and 1980 only resulted in an improvement of biochemical markers of vitamin D deficiency among the children [35].

The benefits of treatment seem clear in our symptomatic vitamin D deficient population, even though we only assessed them by direct questioning and by indirect parameters, as it has been done in other fields [36]. We did not have the set-up to evaluate treatment by costly or invasive methods (dynamometer testing or bone biopsy). The use of vitamin D in Asian immigrants in the UK has been assessed only with biochemical markers [14], demonstrating a response by serum levels of 25-(OH) vitamin D after oral supplementation and no change in the levels of calcium, phosphate, and alkaline phosphatase. But, as we have revealed, hypovitaminosis D concerns a much wider population, including immigrants and non-immigrants, and young and old. One very interesting study showed the effectiveness of an annual megadose of intramuscular cholecalciferol (600,000 IU) in patients with vitamin D deficiency. This therapy appears to be safe, even if certain concerns, such as hypercalciuria, need to be examined. This treatment could potentially be applied on a large scale [37]. Two recent meta-analyses concerning elderly people have shown that intakes of >700 IU are necessary to decrease by approximately 25% (hip, NNT 45/non-vertebral, NNT 27) and 22% (NNT 15) the risk of fractures and falls, respectively [15,16]. The present Recommended Daily Amounts (RDA) in the United States are 200 IU (5 μg) daily for young adults, 400 IU (10 μg) for those aged 51–70, and 600 IU (15 μg) daily for those over 71 years of age [38]. Generally, experts recommend a daily intake of 800–1000 IU per day for concrete benefits in health [39,40]. The mode and frequency of administration remain to be studied in different populations.

In this small group of patients, the parameters studied (age, length of stay in Switzerland, chronic illness, psychiatric co morbidity, veil, and level of 25-(OH) vitamin D were not predictive of response or non-response.

Our study clearly suffers from a number of limitations. First, the study lacks a control group. Second, we do not know the number of patients that were not included in the study because of failure of the physicians to properly record the data. Third, we do not possess the complete biochemical data for all the patients (alkaline phosphatase, PTH, and albumin). Finally, the records can occasionally be imprecise in the descriptions of symptoms and diagnoses.

In conclusion, in the context of resurgent scientific interest in vitamin D, hypovitaminosis D must be considered in a symptomatic, female, asylum-seeking population, to avoid prolonging the duration of chronic symptoms and a potential misdiagnosis of somatisation. The impact of treatment is beneficial, with a rapid resolution of symptoms and reductions in both the use of medical services and the prescription of analgesic drugs. Physicians should therefore be aware of the importance of this disease and the impact of rapid diagnosis and treatment. Future research will have to consider the need for routine supplementation in this and other populations.

**Figure 1:**
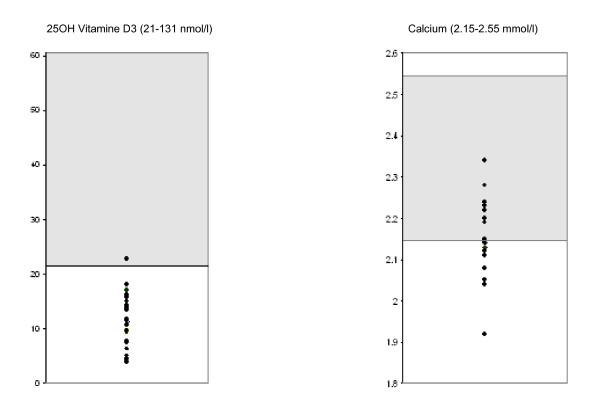
Biochemical results (25-(OH) vitamin D and calcium) of patients at the time of diagnosis.

**Figure 2:**
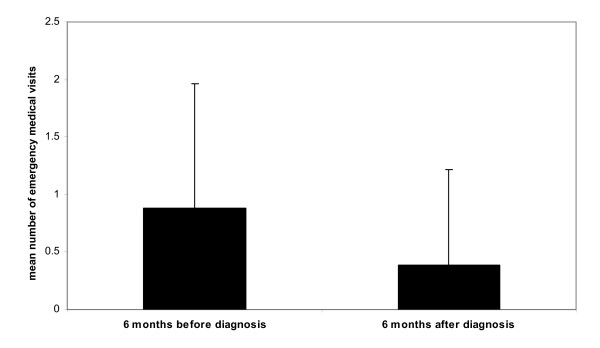
Mean number of emergency medical visits 6 months before and 6 months after diagnosis.

**Figure 3:**
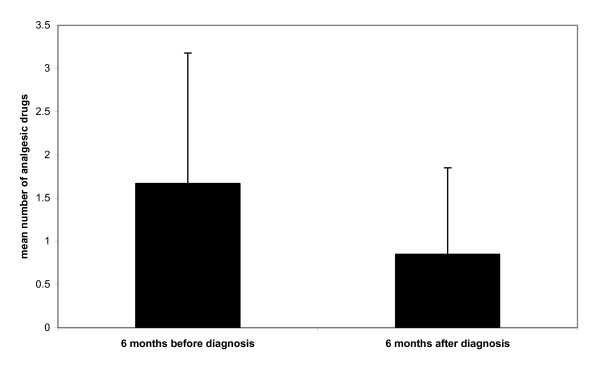
Mean number of analgesic drugs 6 months before and 6 months after diagnosis.

## Pre-publication history

The pre-publication history for this paper can be accessed here:


